# Reinforcement potential of Bioceramic vs. Resin sealers against vertical root fracture risk in the presence of the Butterfly Effect

**DOI:** 10.1016/j.jobcr.2026.101463

**Published:** 2026-05-13

**Authors:** Shailja Paliwal, Anuj Bhardwaj, Surekha Puri Bhat, Pallavi Goyal, Shivani Khandelwal, Dian Agustin Wahjuningrum, Alexander Maniangat Luke, Ayman S. Amer, Ajinkya M. Pawar

**Affiliations:** a Department of Conservative Dentistry & Endodontics, College of Dental Science and Hospital, F12, Jhoomar Ghat, AB Rd, Near Hotel Mashal, Rau, 453331, Indore, Madhya Pradesh, India; b Research Center for Biomaterials and Tissue Engineering. and Department of Conservative Dentistry, Faculty of Dental Medicine, Universitas Airlangga, Surabaya, Jawa Timur, 60132, Indonesia; c Department of Clinical Science, College of Dentistry, Ajman University, Al-Jruf, Ajman, United Arab Emirates; d Centre of Medical and Bio-allied Health Science Research, Ajman University, Al-Jruf, Ajman, United Arab Emirates; e Department of Basic Medical and Dental Sciences, College of Dentistry, Ajman University, Al-Jruf, Ajman, United Arab Emirates; f Department of Conservative Dentistry and Endodontics, Nair Hospital Dental College, Mumbai, Maharashtra, India

**Keywords:** Vertical root fracture, Butterfly effect, Fracture resistance, Calcium-silicate sealer, Dentinal sclerosis, Medicine

## Abstract

**Background:**

Vertical root fracture (VRF) is a major cause of failure in endodontically treated teeth. A distinctive dentinal configuration known as the butterfly effect, characterized by mesiodistal sclerosis, has been linked to increased fracture susceptibility.

**Aim:**

To evaluate the influence of the butterfly effect on fracture resistance of endodontically treated mandibular premolars and to compare the reinforcing potential of root canal sealers.

**Materials and methods:**

Forty-eight freshly extracted single-rooted mandibular premolars were examined under transillumination and classified into teeth with (n = 24) and without (n = 24) the butterfly effect. Each group was further subdivided based on the sealer used for obturation (Bio-C or AH Plus; n = 12 per subgroup). All specimens were instrumented using a standardized rotary protocol and obturated with warm vertical compaction. Fracture resistance was measured under axial loading using a universal testing machine. Data were analysed using two-way analysis of variance (ANOVA) to assess the effects of sealer type and dentinal configuration, followed by independent-samples t-tests for post-hoc comparisons (α = 0.05).

**Results:**

Both dentinal configuration and sealer type affected the fracture resistance (p < 0.001). Teeth without the butterfly effect showed higher resistance than affected teeth, and Bio-C outperformed AH Plus across all subgroups.

**Conclusion:**

Within the limitations of this in vitro study, the results indicate that the butterfly effect adversely affects the post-treatment fracture resistance of roots. Additionally, Bio-C provides a greater level of mechanical support than AH Plus, suggesting a potential biomechanical advantage under the conditions tested.

## 1 Introduction

The goal of root canal treatment is to preserve the tooth by removing the infected pulp and allowing the apical tissues to heal. Although root canal treatment has a high success rate of 93%, crack formation and vertical root fracture [VRF] have recently piqued clinicians' interest and are regarded as critical factors that should not be overlooked because they can result in root canal treatment failure and tooth extraction.^1^

A VRF is defined as a longitudinal fracture that extends in a buccolingual and apical coronal direction toward the apex along the root's long axis. Vertical root fractures remain a principal reason for the loss of endodontically treated teeth, most frequently propagating along the buccolingual plane.^2^ One morphologic feature thought to predispose roots to VRF is the so-called *butterfly effect*, an optical configuration visible in transverse dentine sections.^3^ It has been proposed that teeth with the *butterfly effect* are more likely to develop cracks in this direction due to much increased dentine hardness mesiodistally.^4^

This effect was observed in teeth of various ages and root levels. This pattern shows pronounced mesiodistal sclerosis that outlines two “wings.” Compared to normal dentin, sclerosed dentin is more transparent.^5^ This causes a distinct butterfly shape in transverse parts of the roots due to varying colors of dentine. Sclerotic dentine refracts and scatters light. A decrease in the number of dentinal tubules leads to increased light transmission, resulting in a transparent appearance.^3^

**Fig. 1:**
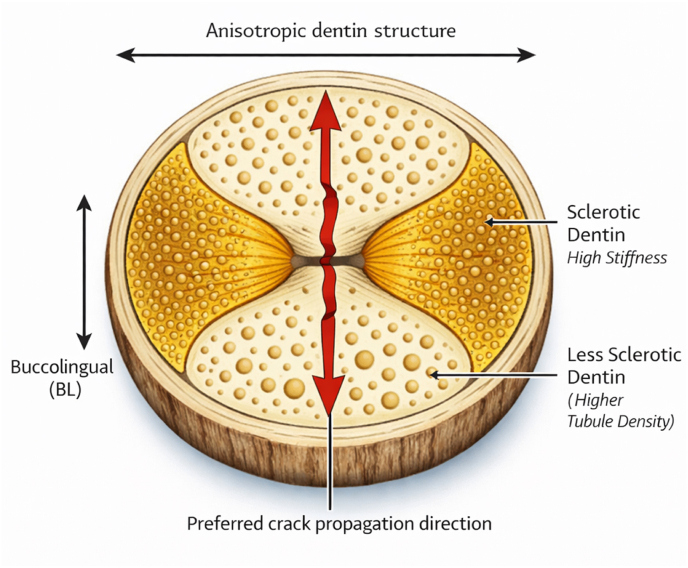
Schematic representation of the butterfly dentin configuration in root cross-section. Mesiodistal regions exhibit increased dentinal sclerosis and higher stiffness, while buccolingual regions show relatively higher tubule density and reduced mineralization. This anisotropic structural organization promotes preferential stress concentration and crack propagation along the buccolingual direction.

Microscopic studies attribute the image to regional differences in tubule orientation and density: tubules are more numerous buccolingually and markedly sclerotic mesiodistally, producing zones with disparate mechanical behaviour. ^6^Biologically, the morphology of a butterfly configuration exhibits the structural anisotropy of the dentin, wherein the mesiodistally oriented sclerotic structures will have an increase in mineral density and stiffness, as compared to the buccolingually oriented structures which are less sclerotic, thus maintaining a greater density of tubules and a higher degree of permeability. The resultant anisotropic condition will lead to non-uniform elastic moduli and distribution of strain in response to functional loading, thus contributing to stress concentrations occurring more commonly along the buccolingual loading and facilitating the preferential initiation and propagation of cracks. In addition, the anisotropic stress response may have primary implications for predisposition of roots to vertical fractures, as well as affecting how restorative materials interact with the underlying dentin. Such heterogeneity may influence not only the fracture threshold of dentine but also its interface with root-canal sealers.^6^ The structural orientation and biomechanical implications of the butterfly dentin configuration are illustrated schematically in Fig. 1. The relationship between dentinal configuration and the preferential direction of fracture propagation is summarized in Table 1.

**Table 1:** Relationship between dentinal configuration and fracture behavior.

Dentinal Configuration	Structural Characteristics	Mechanical Behavior	Preferred Fracture Direction	Clinical Implication
Butterfly dentin	Increased mesiodistal sclerosis; reduced tubule density in MD direction	Anisotropic stiffness; stress concentration in weaker regions	Buccolingual direction	Higher susceptibility to vertical root fracture
Non-butterfly dentin	Relatively uniform tubule distribution	More homogeneous stress distribution	No dominant fracture pattern	Greater resistance to fracture

A sealer's clinical role is to create an impervious three-dimensional fill, penetrating patent tubules, blocking voids and isolating residual microorganisms.^7^ Penetration depth and interfacial adaptation, however, depend on factors like tubule calibre, patency and the degree of sclerosis—variables that the butterfly effect could alter substantially.^8^ Resin-based and calcium-silicate [bioceramic] sealers exhibit divergent flow, setting reactions and chemical affinity for dentine. Understanding how each material interacts with butterfly-pattern versus non-butterfly dentine is vital for optimising obturation quality and may provide improved mechanical performance under experimental conditions.^9^

Prior research has highlighted the biomechanical significance of the butterfly dentin configuration, mostly by conducting studies on dentinal microhardness, crack formation, and fracture line propagation. For example, researchers have found that teeth with this configuration may have increased mesiodistal hardness than those with other dentin shapes, demonstrating an anisotropic behaviour of the dentin, which has increased the likelihood of fractures propagating in the buccolingual direction.^3^^,^^4^ Additionally, when attempting physiological instrumentation on teeth with a butterfly configuration, investigators have shown that this dentin type is more susceptible to structural damage due to crack formation when compared to other dentin shapes.^10^ However, previous studies have only reported descriptive accounts or only examined the fracture resistance of butterfly dentin without integrating this type of dentin into a common model of standardised fracture resistance for all types of dentin. In particular, little to no work has been conducted to date examining the interaction of contemporary root canal sealers and the butterfly pattern of dentin using controlled mechanical testing. Therefore, the objective of this present study is to investigate the butterfly dentin configuration in a controlled fracture resistance model for a comprehensive understanding of the mechanical behaviour of this anatomical variant and how it interacts with various resin sealer chemistries.

Therefore, the present in-vitro study was designed to evaluate the influence of butterfly dentin configuration on fracture resistance and to investigate the interaction between dentinal morphology and sealer type, specifically comparing a calcium–silicate bioceramic sealer (Bio-C) and an epoxy resin-based sealer (AH Plus). The null hypotheses tested were: (1) there is no significant difference in fracture resistance between teeth with and without the butterfly dentin configuration; (2) there is no significant difference in fracture resistance between teeth obturated with bioceramic and epoxy resin-based sealers; and (3) there is no interaction effect between dentinal configuration and sealer type on fracture resistance.

## 2 Methods

### 2.1 Study design and tooth collection

Ethical approval for the use of extracted human teeth was obtained from the Institutional Ethics Committee of College of Dental Sciences and Hospital (reference no. CDSH/Admin/2025 dated 06/10/2025). An in-vitro, four-arm comparative investigation was performed on single-rooted mandibular premolars removed for orthodontic therapy. Immediately after extraction, each specimen was freed of adherent tissue and calculus with an ultrasonic scaler, then submerged in sterile isotonic saline at 4 °C. Storage fluid was renewed every 48 h to inhibit microbial growth and preserve dentinal moisture. A stereomicroscope [ × 20] was used to screen for cracks, restorative work, caries, resorption defects, open apices, or previous endodontic intervention; teeth showing any of these conditions were discarded. Only roots with a single straight canal [<5° curvature] advanced to the experimental phase.

### 2.2 Sample size calculation

Sample size was estimated in G∗Power 3.1.9.4 using a fixed-effects one-way ANOVA model with four groups, assuming a large effect size (f = 0.50), α = 0.05, and 1–β = 0.80, which yielded a required minimum sample size of 48 specimens (n = 12 per group).

### 2.3 Standardisation and group allocation

**Fig. 2:**
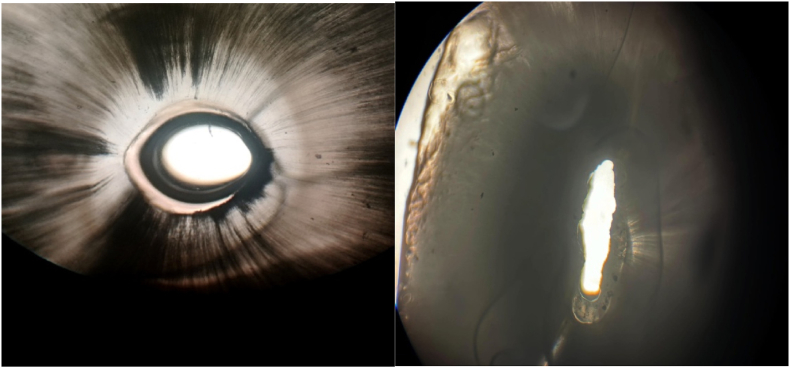
Teeth exhibiting butterfly effect and those without, observed under a light microscope at 10 × magnification.

Crowns were removed at the cemento-enamel junction with a water-cooled diamond disc. To obtain a uniform section, an additional 1 mm of dentine was trimmed. Each specimen was secured on a glass slide and transilluminated [Fig. 2] to identify the *butterfly effect*—a mesiodistal opacity pattern. Twenty-four roots displaying the pattern were assigned to **Group A**, whereas twenty-four without it formed **Group B**. A computer-generated randomization list then divided both cohorts into two equal subgroups according to the sealer planned for obtu ration. Computerized randomization through a randomization website (www.randomization.com) was used for sample selection. To conceal allocation, samples were placed in numbered opaque containers according to their assigned group prior to being allocated. An independent operator who had no involvement with the preparation or obturation of specimens, nor in the evaluation of outcomes was used for the entire allocation process, reducing the potential for bias due to selection. All specimens were subjected to a standardized preparation and obturation protocol to ensure uniformity across experimental groups and to minimize procedural variability.

• **Group A1:** Butterfly + Bio-C [bioceramic; Angelus, Londrina, Brazil]
• **Group A2:** Butterfly + AH Plus® [epoxy resin; Dentsply DeTrey, Konstanz, Germany]
• **Group B1:** No-butterfly + Bio-C
• **Group B2:** No-butterfly + AH Plus®

Thus, each of the four experimental arms contained twelve specimens.

The sealer made from calcium silicate (Bio-C, Angelus, Londrina/Brasil) is mostly made up of three main components (tricalcium silicate and dicalcium silicate) to form a compound consisting of these two compounds together with several other materials (calcium hydroxide, calcium oxide, zirconium oxide (as a radiopaque agent), and thickening agents). The sealer made from resin is made from two main epoxy resins (bisphenol-A epoxy resin and bisphenol-F epoxy resin) along with various additional constituents (calcium tungstate, zirconium oxide, silica and iron oxide pigment). The exact mixing ratios of these materials are not provided in the manufacturer's specifications. Both sealers used in this study were supplied in premixed or paste–paste formulations; therefore, powder–liquid ratios were not applicable. Bio-C is a ready-to-use premixed bioceramic sealer, while AH Plus is provided as a two-paste system mixed according to the manufacturer's instructions to achieve a homogeneous consistency.

### 2.4 Canal preparation and obturation

Working length [WL] was established by inserting a #10 K-file [Dentsply Maillefer, Ballaigues, Switzerland] until its tip became visible at the major apical foramen, then withdrawing 1 mm. Rotary shaping was carried out with ProTaper Gold instruments (Dentsply Maillefer, Ballaigues, Switzerland) to size F2 [25/08]. Between each instrument, canals were irrigated with 3 mL of 5.25% sodium hypochlorite (NaOCl; Prime Dental, Mumbai, India) using a 30-gauge side-vented needle, resulting in an approximate total NaOCl volume of 15–18 mL per canal during instrumentation. Following canal preparation, a final rinse was performed using 5 mL of 17% ethylenediaminetetraacetic acid (EDTA; DentWash; Prime Dental PVT., Mumbai, India) for 2 min to remove the smear layer, followed by a final flush with 5 mL of physiological saline.

**Fig. 3:**
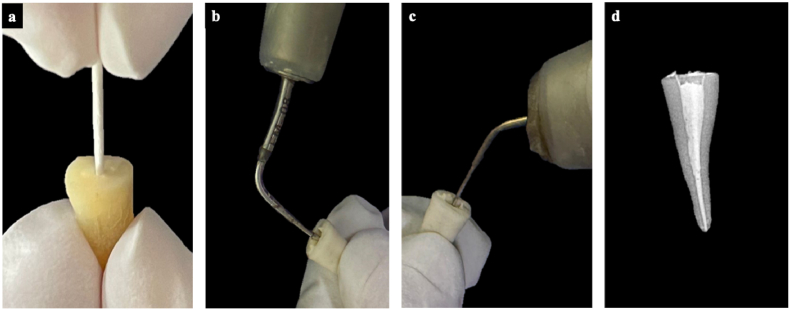
**Representative steps of the obturation procedure using warm vertical compaction technique.** (a) Master gutta-percha cone coated with sealer and positioned to working length, (b) Down-pack phase using System B heat source for vertical compaction of gutta-percha, (c) Backfilling of the canal with thermoplasticized gutta-percha to achieve a three-dimensional seal, and (d) Post-obturation radiograph demonstrating the final quality of root canal filling.

Canals were dried using sterile absorbent paper points until complete dryness was achieved, as confirmed by the absence of moisture on the final paper point before initiating obturation. For obturation, an F2-taper gutta-percha [Dentsply Maillefer, Ballaigues, Switzerland] cone was lightly coated with the allocated sealer and seated to WL. Warm vertical compaction was performed with System B pluggers (System B obturation unit; Kerr Endodontics, Orange, CA, USA), and backfilling was completed with thermoplasticised gutta-percha. The sequential steps of the obturation procedure are illustrated in Fig. 3. The access was sealed provisionally with Cavit G [3M ESPE, Seefeld, Germany]. All specimens were stored at 37 °C and 100% relative humidity for 14 days to allow complete setting of the sealers. These conditions were selected to simulate the intraoral environment and to ensure optimal hydration and maturation of calcium–silicate-based materials, as recommended in previous studies evaluating the physicochemical and mechanical properties of endodontic sealers^11^^,^^12^

An experienced single operator completed all procedures therefore reducing variability between operators. Prior to initiating the study, the operator had been standardised through pilot procedures for consistent canal preparation, irrigation and obturation protocols. Standardised working length determination, instrument sequencing, irrigation volume and obturation method were strictly followed during the course of the study to ensure consistent procedural outcomes.

### 2.5 Fracture-resistance assay

After the curing period, each root was embedded in self-curing acrylic within a cylindrical PVC mould, leaving 2 mm of the coronal root exposed to mimic alveolar bone height. A 0.2-mm layer of silicone impression material lined the socket to simulate a periodontal ligament and minimize stress concentration. The acrylic block was clamped in a custom steel jig affixed to the lower platen of a universal testing machine (Instron KIC 2100 C, Instron Corp., Norwood, MA, USA). A hardened-steel indenter [3 mm diameter] attached to the cross-head contacted the root canal orifice along the long axis. Compressive load was applied at 1 mm min^−1^ until catastrophic fracture, defined by a sudden ≥25 % drop in the load–displacement curve. A crosshead speed of 1 mm/min was selected in accordance with commonly used protocols for static fracture resistance testing of endodontically treated teeth, as it provides controlled and reproducible load application under in vitro conditions. The maximum force in newtons was automatically recorded for each specimen. The operator performing the fracture resistance testing was blinded to group allocation to minimize potential measurement bias. The maximum force in newtons was automatically recorded for each specimen. The operator performing the fracture resistance testing was blinded to group allocation to minimize measurement bias.

### 2.6 Statistical analysis

Assumptions for two-way ANOVA were assessed before analysis. Normality of model residuals was evaluated using the Shapiro–Wilk test, and homogeneity of variance across groups was assessed using Levene's test. The ANOVA for fracture resistance from different types of sealers (Bio-C vs AH Plus) and dentinal configurations (butterfly vs non-butterfly) was conducted by Two-Way ANOVA. The individual and interactive effects of type of sealer and type of dentin configuration on fracture resistance were examined. Independent sample t-tests were used for post-hoc pairwise comparisons. A full factorial two-way ANOVA model including both main effects and their interaction was prespecified a priori based on the study objectives. No model-based variable selection or stepwise elimination procedure was performed. All statistical analysis was performed with IBM SPSS version 25 software and as stated above at an alpha level of 0.05.

## 3 Results

Operator calibration demonstrated high procedural consistency, with repeated pilot assessments showing minimal variability across key steps of canal preparation and obturation. The intra-operator agreement was high (intraclass correlation coefficient [ICC] = 0.94), indicating excellent reproducibility of the standardized protocol.

The results of fracture resistance testing for all groups (B1 through A2) are summarized in Table 2. The highest fracture resistance was associated with the non-butterfly (B1) Bio-C group and exhibited a mean value of 221.08 N (standard error = 4.52 N, 95% CI = 218.21 – 223.96 N), whereas the lowest fracture resistance values were exhibited by the butterfly (A2) AH Plus group (mean = 147.00 N ± 6.34 N, 95% CI = 142.97 – 151.03 N). Intermediate fracture resistance was demonstrated by the butterfly (A1) Bio-C group (mean = 185.75 N ± 4.31 N; 95% CI = 183.01 – 188.49 N) and the non-butterfly (B2) AH Plus group (mean = 169.67 N ± 5.30 N; 95% CI = 166.30 – 173.03 N). The lack of overlap in the confidence intervals between the groups indicates that these groups are clearly separated biomechanically.

**Table 2:** Descriptive statistics of fracture resistance (N) across experimental groups (n = 12 per group).

Group	Dentinal Condition	Sealer Type	n	Mean ± SD (N)	95% CI (N)
A1	Butterfly	Bio-C	12	185.75 ± 4.31	183.01–188.49
A2	Butterfly	AH Plus	12	147.00 ± 6.34	142.97–151.03
B1	Non-butterfly	Bio-C	12	221.08 ± 4.52	218.21–223.96
B2	Non-butterfly	AH Plus	12	169.67 ± 5.30	166.30–173.03

**Fig. 4:**
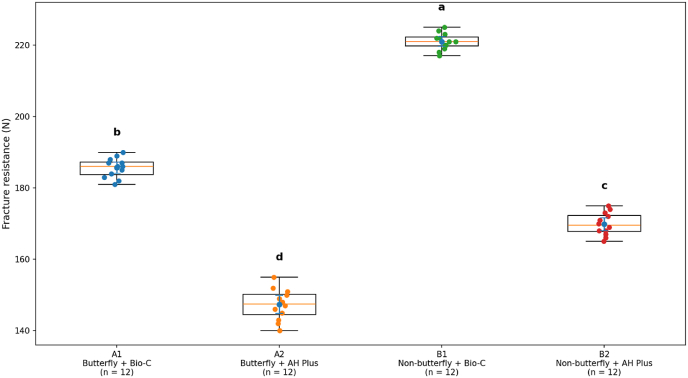
Distribution of fracture resistance values across experimental groups. Individual data points are shown along with boxplots representing data distribution. Mean values are indicated with markers, and error bars represent 95% confidence intervals. Each group consisted of n = 12 specimens. Different lowercase letters indicate statistically significant differences between groups (P < 0.05).

Fig. 4 presents the spread and variability of the fracture resistance values among each group and the individual data point, mean, and measure of dispersion are included. The figure demonstrates that the groups are evenly distributed and clearly show that the Bio-C sealer had superior performance compared to each other in these two conditions of dentin.

Prior to inferential analysis, assumptions for two-way ANOVA were assessed. The residuals were checked using the Shapiro–Wilk test (W = 0.960, P = 0.097) and were normally distributed. Levene's test showed homogeneity of variance (F = 0.369, P = 0.776), which confirms that it is appropriate to conduct parametric testing. The summary data of the two-way ANOVA are contained in Table 3. There was a very large main effect for sealer type (F(1,44) = 909.59, P = 4.98 × 10^−31^) with η^2^ = 0.954; this shows that the type of sealer used has a major impact on the variability of fracture resistance. The dentinal configuration also produced a significant main effect (F(1,44) = 376.37, P = 3.44 × 10^−23^) with an effect size of η^2^ = 0.895; therefore, the butterfly configuration of dentin has a significant negative effect on structural resistance.

**Table 3:** Two-way ANOVA summary for the effects of sealer type and butterfly effect on fracture resistance.

Source of Variation	df	Sum of Squares	Mean Square	F value	Exact P
Sealer type	1	24390.08	24390.08	909.59	4.98 × 10^−31^
Dentinal configuration	1	10092.00	10092.00	376.37	3.44 × 10^−23^
Sealer × Configuration	1	481.33	481.33	17.95	0.000114
Error	44	1179.83	26.81	—	—
Total	47	36143.25	—	—	—

∗Two-way ANOVA. p < 0.05 considered statistically significant.

A significant interaction was observed between dentinal configuration and type of sealer (F(1,44) = 17.95, P = 0.000114; partial η^2^ = 0.290) as outlined in Table 2, indicating that the type of sealer has different levels of reinforcing ability based on the underlying dentin morphology. Most notably, while Bio-C has a high degree of fracture resistance compared with AH Plus, its ability to reinforce was greater in the case of roots that did not have a butterfly-shaped dentin configuration.

When arranging the groups in order of fracture resistance, the following rank order was established: B1 (Non-butterfly + Bio-C) > A1 (Butterfly + Bio-C) > B2 (Non-butterfly + AH Plus) > A2 (Butterfly + AH Plus). Therefore both type of sealer and dentin architecture work independently and interactively to affect biomechanical properties of dentin.

## 4 Discussion

Teeth that have been endodontically treated lose a significant amount of dentin during treatment as well as have a decreased risk of fracture; therefore, they are more prone to fracture than vital teeth. Vertical root fractures (VRF) are associated with a poor prognosis since the extraction of the tooth or root amputation may be necessary after VRF, which will negatively affect the long-term prognosis for the tooth.^2^^,^^13^ Many studies have shown that instances of VRF are significantly more common in endodontically treated teeth than in untreated teeth. It is essential to identify materials that optimize the mechanical performance for the area where the fracturing will take place in order to create optimal mechanical strength of both the apex and the frenulum following treatment.^14^^,^^15^ Considering this, the purpose of the current study was to evaluate how dentinal configuration and the properties of the sealing agent will influence the mechanical strength of endodontically treated teeth under laboratory conditions.

The butterfly shape of the dentin configuration is an example of a structurally different type of dentin that has significantly enhanced mesiodistal sclerotic regions, and lesser buccolingual mineralized regions. The anisotropic nature of this structure creates variability in dentin characteristics: differences in tubule density, mineral content, and elastic modulus.^16^ This structural difference creates an uneven stress distribution when functionally loaded, therefore increasing the potential for roots to crack at or near the buccolingual axis. Previous investigations found that fractures in teeth exhibiting the butterfly effect primarily occur along this axis, consistent with the pattern of dentin sclerotic changes observed. Findings from this study support these findings as teeth with the butterfly configuration have significantly less fracture resistance than those without this structural configuration.^17^^,^^18^ Thus supporting the hypothesis that dentin anisotropy is an important factor in determining the biomechanical behavior of endodontically treated roots.

The selection of mandibular premolars in this study is clinically relevant, since the teeth have higher functional stress associated with their anatomy such as having a smaller mesiodistal dimension and having an oval shaped buccolingual canal configuration.^19^ Due to their anatomical characteristics, mandibular premolar teeth may be more likely to fracture as a result of axial loading. By using standardized experimental conditions (e.g., canal preparation, irrigation, obturation protocols), this study was designed to provide an isolated evaluation of the influence of dentinal morphology and sealer type on fracture resistance, enhancing the internal validity of the data.^20^

The results further demonstrated that sealer type significantly influences fracture resistance due to being either calcium silicate or epoxy resin based also produced different amounts of fracture resistant dentin regardless of where the fracture happens underneath. The findings of the present study indicate that sealer composition plays a critical role in influencing fracture resistance, irrespective of the underlying dentinal configuration.

Superior performance of Calcium-silicate sealers can be attributed to their unique physicochemical properties and bioactive characteristics. Calcium silicate materials differ from resin-based sealers in that they are inherently hydrophilic, and therefore can create favorable interactions with moisture in the intraradicular environment, and, when setting, calcium silicate materials will undergo a hydration reaction leading to the creation of calcium hydroxide, and subsequently precipitating hydroxyapatite at the sealer/dentin interface.^9^^,^^21^ In turn, this biomineralization creates a mineralized layering at the interface, improving the continuity of the dentin to the sealer and gutta-percha for more homogeneous interfaces and better compatibility for transferring mechanical loading.^22^

In addition, both calcium ion release during the setting reaction and alkaline conditions promote both improved interface adaptation and sealing ability.^23^ The fine particle size and low viscosity of calcium-silicate sealers help promote deeper penetration into the dentin tubules and establish a three-dimensional interlocking network that increases micromechanical retention.^24^ In combination, these properties can help improve stress redistribution across the sealer-dentin interface so as to reduce localized stress concentration, thereby delaying the initiation of cracks. The present study confirms this mechanism, as Bio-C had a superior fracture resistance to AH Plus for all of the experimental groups.

Rather than using micromechanical retention and chemical bonding alone, the sealers based on epoxy resins like AH Plus depend greatly upon several forms of chemical interactions with the dentin surface. Although these sealers have been popular because they are easy to use, maintain their dimensional stability over time, and do not shrink significantly after hardening, they can become less effective if moisture remains behind in the root canal area where the sealing occurs.^25^ There may also be potential areas of concern with regards to the ability of these sealers to provide adequate bonding around the larger sections of the root canals. This is due in part to the possibility that incomplete polymerization will create voids or varying thickness of material between the seals and the dentin surface creating structural discontinuities that can increase the likelihood of failure when loads are applied. Consequently, the relatively lower fracture resistance noted with respect to the AH Plus groups in the present study is consistent with other studies that have reported similar outcomes with all types of resin-based sealers under wet conditions.^26^

One of the main strengths of this study is that it uses a controlled mechanical loading procedure, which minimizes the effects of confounders and provides a means of evaluating fracture resistance with precision and reproducibility. All specimens were standardized during preparation, all canals were instrumented uniformly, and all obturation techniques were identical, increasing the ability to draw reliable conclusions. However, these findings must be interpreted in light of the limitations associated with in vitro experimentation.

The mechanical tests conducted in this report were done using static vertical loads, which does not entirely mimic the usual dynamic and cyclic forces on the teeth that occur during chewing. Teeth are subject to very complex load patterns: cyclic fatigue, lateral (side-to-side) forces, and thermal (temperature) fluctuations in the mouth. All of these forces can affect how fractures occur. Additionally, because the authors did not use aging tests (such as thermocycling or applying heat and cold) on the materials before conducting the mechanical tests, the authors are not able to replicate long-term clinical conditions or how the material has degraded over time. Furthermore, the authors were not able to evaluate in detail how cracks begin and progress through the tooth root structure because they did not utilize micro-CT (micro-focused computed tomography) in their analysis.

Although appropriate statistical methods were employed to establish sample size for each subgroup, the relatively small sample size, as well as only using one tooth type (mandibular premolars) limits the ability to generalize findings from these studies to other tooth types or morphology with differing anatomy and biomechanics making this measure of fracture resistance an indirect measure of the mechanical outcome of the method tested and will not serve as a direct predictor for clinical success.

### 4.1 Clinical implication

The butterfly shape of the dentin arrangement could be important in identifying teeth that are more likely to break. Teeth that possess mesiodistal sclerosis and its related anisotropic nature appear to have an increased risk of developing vertical root fractures when loaded. Based on the results of this study, utilizing calcium–silicate bioactive and hydrophilic sealers can enhance mechanical properties at an experimental level when the dentin is structurally impaired. Although calcium–silicate sealers may contribute to improved fracture resistance, there are other factors that also play a role in the long-term prognosis of endodontically treated teeth, which include occlusal forces, ferrule design, restorative material choice, and patient factors. Thus, although calcium–silicate sealers may facilitate improved resistance to fracture, the need for a comprehensive approach to treatment planning is imperative in achieving success in the long-term outcome of endodontically treated teeth.

Overall, results from this research show that two factors affect the fracture resistance of endodontically (root-canal) treated teeth: dentinal architecture and the cement used to seal the root. The butterfly configuration of dentin reduces the strength of the tooth, which makes it more likely that the tooth will fracture. The use of calcium silicate cements enhances the fracture resistance of endodontically treated teeth by providing better adaptation to the tooth structure and redistributing stress; thus, the findings improve our understanding of the biomechanical behavior of endodontically treated teeth and highlight the need for consideration of both anatomical (i.e., anatomical tooth structure) and material (i.e., sealing cement) factors in clinical treatment decisions.

## 5 Conclusion

Within the limitations of this in vitro study, the butterfly dentin configuration was associated with reduced fracture resistance. The calcium–silicate bioceramic sealer Bio-C demonstrated higher fracture resistance compared to the epoxy resin-based sealer AH Plus under both dentinal conditions. These findings suggest a potential biomechanical advantage; however, fracture resistance outcomes should not be directly extrapolated to clinical survival, and further clinical studies are required to validate their clinical relevance.

## Patient/guardian consent

Invitro study design. Not applicable for this study.

## Ethical statement

Ethical clearance for the procurement and utilization of freshly extracted human teeth was formally granted by the Institutional Ethics Committee (IEC) of the College of Dental Sciences and Hospital, verifying complete institutional oversight (Reference No. CDSH/Admin/2025, dated October 6, 2025).

## Source(s) of support

This research is collaborated research funded by the Indonesian Endowment Fund for Education (LPDP) on behalf of the Indonesian Ministry of Higher Education, Science and Technology and managed under the EQUITY Program (Contract No. 4300/B3/DT.03.08/2025, No. 297/UN3/HK.07.00/2025) and International Research Collaboration Top Tier Scheme No. 5442/B/UN3.LPPM/PT.01.03/2025.

## Declaration of competing interest

The authors declare that they have no known competing financial interests or personal relationships that could have appeared to influence the work reported in this paper.
